# Translational Database Selection and Multiplexed Sequence Capture for Up Front Filtering of Reliable Breast Cancer Biomarker Candidates

**DOI:** 10.1371/journal.pone.0020794

**Published:** 2011-06-15

**Authors:** Patrik L. Ståhl, Magnus K. Bjursell, Hovsep Mahdessian, Sophia Hober, Karin Jirström, Joakim Lundeberg

**Affiliations:** 1 Science for Life Laboratory, Division of Gene Technology, Royal Institute of Technology, Solna, Sweden; 2 Science for Life Laboratory, Department of Molecular Medicine and Surgery, Karolinska Institutet, Solna, Sweden; 3 Division of Proteomics, School of Biotechnology, AlbaNova University Center, Royal Institute of Technology, Stockholm, Sweden; 4 Division of Pathology, Department of Clinical Sciences, Skåne University Hospital, Lund University, Lund, Sweden; University of South Florida College of Medicine, United States of America

## Abstract

Biomarker identification is of utmost importance for the development of novel diagnostics and therapeutics. Here we make use of a translational database selection strategy, utilizing data from the Human Protein Atlas (HPA) on differentially expressed protein patterns in healthy and breast cancer tissues as a means to filter out potential biomarkers for underlying genetic causatives of the disease. DNA was isolated from ten breast cancer biopsies, and the protein coding and flanking non-coding genomic regions corresponding to the selected proteins were extracted in a multiplexed format from the samples using a single DNA sequence capture array. Deep sequencing revealed an even enrichment of the multiplexed samples and a great variation of genetic alterations in the tumors of the sampled individuals. Benefiting from the upstream filtering method, the final set of biomarker candidates could be completely verified through bidirectional Sanger sequencing, revealing a 40 percent false positive rate despite high read coverage. Of the variants encountered in translated regions, nine novel non-synonymous variations were identified and verified, two of which were present in more than one of the ten tumor samples.

## Introduction

Discovery of biomarkers has traditionally been mediated by interpretation of transcriptome data generated using array-based expression profiling platforms [1], [2]. This has for instance resulted in better understanding of prostate cancers, where the initial screening for serum PSA has led to earlier detection of the disease, but also to inaccurate diagnosis, necessitating discovery of new and better biomarkers [3], [4]. It has earlier been shown that biomarker discovery benefits from integration of genomic and proteomic technologies [5]. Recent data have also demonstrated a stronger correlation between transcripts and proteins than previously anticipated [6], [7]. Thus, translating aberrant protein expression patterns to transcript differences is of high interest, and the possibility that some of these changes are encoded in the genome offers new openings to identify causative mutations. Today's novel technologies and large-scale efforts for proteomic screening are providing the grounds for a great increase of pace in such biomarker discovery. The Human Protein Atlas is one example where the proteome is being screened for differences in expression patterns in a large collection of cancers and corresponding healthy tissues [8], [9]. Antibodies are created for each human protein in a mono-specific polyclonal fashion and are used to stain tissue microarrays in order to determine the expression patterns and levels.

In addition to more efficient proteomic screening, the advent of massively parallel sequencing methods [10], [11], [12] has greatly increased the throughput of genomic data [13] and has also provided new platforms for transcriptomic screening past the traditional array based technologies [14], [15]. Whereas genomes of healthy and diseased tissues can be sequenced in full today [16], [17], the sequencing throughput is still not able to provide us with enough data to screen vast numbers of genomes in parallel in a cost-efficient manner. The response to this has traditionally been selection of genomic regions of interest by PCR [18], but has lately been replaced by methods for extraction of regions of interest by sequence enrichment strategies [19], [20], [21].

Here we demonstrate a model for further cost reduction and increased efficiency in biomarker discovery by employing up-front database selection in combination with sample barcoding [22] and multiplexed sequence capture enrichment, to rationally filter out potential biomarkers at an early stage.

## Materials and Methods

### Ethics statement

Ethical permission was obtained from the Ethics Committee at Lund University whereby informed consent was deemed not to be required other than by the opt-out method. The study was conducted according to Declaration of Helsinki Principles. The data were analyzed anonymously.

### Translational database selection

The Human Protein Atlas database [8] was searched for proteins with a clearly differential staining pattern in healthy breast tissue and breast cancer tissue requiring: i) breast glandular cells with no staining and ii) breast cancer tumor cells with at least five patients with strong staining. A complementary search for breast glandular cells with strong staining and breast cancer tumor cells with at least ten patients with weak or no staining was also carried out.

Further, the proteins found through the search results were screened to match a number of criteria of interest. These included overall differential staining of healthy and cancerous tissues for the particular protein and high assay validation scores. Proteins were scored as particularly interesting if they were present in a transmembrane region, and if they contained a signaling peptide. In total, 41 proteins were selected in this way and an additional 10 proteins known to be associated with cancer from the literature were added to the list. (Table S1)

### Selection and design of regions for genomic enrichment

The coding exons for the proteins selected through the HPA database were extracted from the UCSC human reference genome (hg18) database. Additionally the 5′UTR and 3′UTR regions were included, as well as 1000 basepairs upstream from the 5′UTR. To facilitate efficient capture of the targeted genomic regions, the selected regions were expanded to a minimum of 250 basepairs and regions with a resulting overlap were fused together. 479 regions totaling 303,788 basepairs, 89,705 of which were protein coding, were selected in this way.

The selected regions were submitted to the array manufacturer (Nimblegen, Madison, WI, USA) for manufacturing of 385k-feature enrichment arrays. The final design after internal processing and filtering of repetitive regions contained 581 tiled regions spanning a total of 303,986 target bases.

### Sampling of tumors and DNA extraction

Tumors were surgically removed from the patients, trimmed for healthy tissue and instantly put into a freezer at −20°C. >99% of the cells were judged to be of tumor origin. For extraction of DNA ten pieces approximately 1 mm^3^ each were cut out from each tumor and put into a fastprep tube (164102930, Lysing matrix D, Fisher Scientific, Gothenburg, Sweden). 360 µl of ATL buffer from the DNeasy (Qiagen, Valencia, CA, USA) kit was added to the tube that was then processed 2 times 60 seconds on a Fastprep FP210 system (Qbiogene, Carlsbad, CA, USA). The homogenized liquid phase was pipetted into a Qiashredder column (Qiagen, Valencia, CA, USA) that was centrifuged for 1 minute at 13k rpm. 40 µl of proteinase K (Qiagen, DNeasy kit) was added to the shredded material followed by a 15-minute incubation of the sample at 56°C. 300 µl of buffer AL (DNeasy, Qiagen) and 300 µl 96% ethanol was added to the sample and the resulting mixture was split in half and transferred into 2 DNeasy Mini spin columns. The columns were processed according to manufacturers instructions (DNeasy, Qiagen) after which each sample was eluted twice with 200 µl of EB buffer (DNeasy, Qiagen) in separate tubes, totaling 4 tubes with 200 µl eluate each for each initial tumor sample.

Following elution each sample was ethanol precipitated by adding 20 µl 3 M NaAc and 500 µl −20°C 96% ethanol, and then incubated at −80°C for 15 minutes. After freezing the sample was centrifuged at 13k rpm for 25 minutes in room temperature. The sample was washed with 500 µl −20°C 70% ethanol and centrifuged at 13k rpm for another 15 minutes in room temperature. The liquid was removed and the sample tubes were dried at room temperature over night with open lids under a protective cover. The following day 20 µl 1xTE buffer was added to each sample tube to dissolve the extracted DNA after which the tubes for each sample were pooled and analyzed for concentration and purity on a NanoDrop N-1000 spectrophotometer (NanoDrop, Wilmington, DE, USA).

Healthy reference tissue from each patient sample was processed in the same way to obtain DNA for validation sequencing of variations indicative of potential biomarkers.

### DNA enrichment and sequencing

A total of ten DNA samples from breast cancer tumors were processed into sequencing libraries using an in house developed automated protocol [23] based on the GS FLX titanium Library preparation method (Roche/454, Branford, CT, USA) using multiplex identifier handles (MID 1–10, Roche/454). The samples were then pooled in equimolar ratios into a single tube and enriched for target sequences by hybridizing to the custom Nimblegen 385k array (Roche/Nimblegen, Madison, WI, USA) previously designed and manufactured for the project. Following enrichment the pooled library was titrated and sequenced according to manufacturers instructions (Roche/454) on a GS FLX using long-read titanium chemistry.

### Data analysis

The data corresponding to each sample was mapped to the human reference genome (hg19) using the Roche/454 GS Mapper software (Newbler version 2.3), and the file containing the resulting high confidence variants (HCdiffs.txt) was used for further analysis. The single nucleotide variants were annotated using custom perl scripts and the knownGene transcript database (UCSC). The variants were sorted by the number of samples they were present in. Information from dbSNP 130 was used to extract previous knowledge about specific SNPs.

### Validation of variations

Following Roche/454 sequencing and data analysis as described above, 15 novel non-synonymous variations were encountered, spread across the different samples (Table S2). These were verified through amplification by PCR followed by bidirectional Sanger sequencing of the corresponding genomic regions. Nine out of these variations were confirmed as heterozygous in both tumor tissue and healthy reference tissue, whereas six of the variants were not visible in either tissue in the validation experiments, hence deemed false positives.

## Results

**Figure 1:**
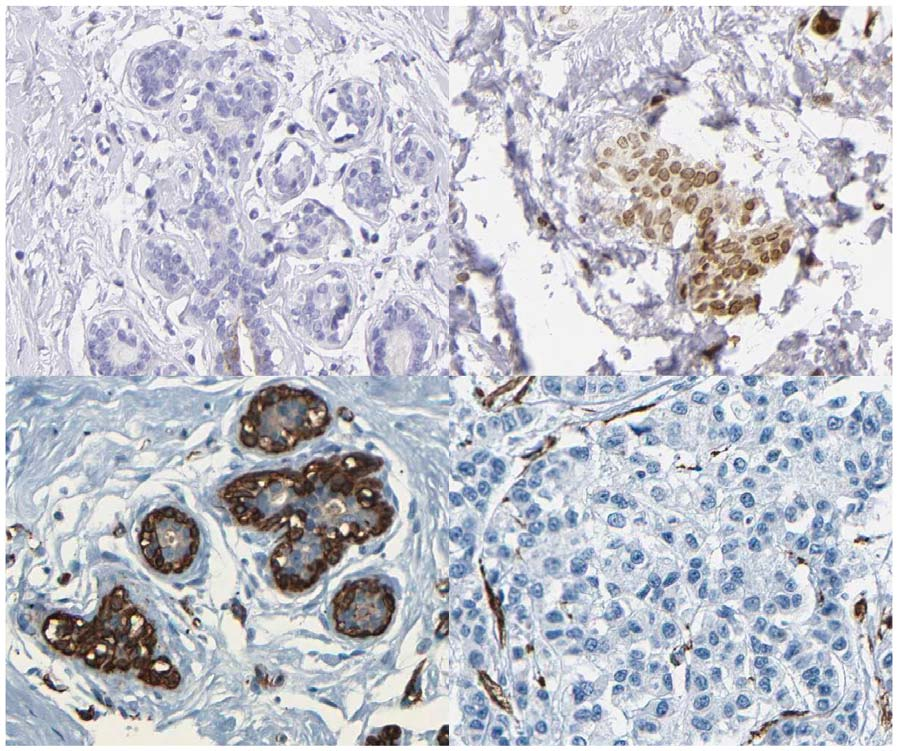
Principle of a differentially expressed protein, as seen on the tissue array images in the Human Protein Atlas database. Immunohistochemical staining using antibodies targeting the interrogated protein in the tissue sectionss gives rise to a localized and distinct brown color. In the first case (top) the healthy breast tissue does not seem to show any expression of the PIP protein, whereas the breast cancer tissue shows heavy expression of the targeted protein. In the second case (bottom) the healthy breast tissue shows heavy expression of the ACTG1 protein, whereas the breast cancer tissue does not seem to show any expression of the targeted protein.

DNA from breast cancer samples and surrounding healthy breast tissue (Figure 1) was isolated from ten surgically removed tumors and selected genes were investigated using array-based enrichment and DNA sequencing. In total, 581 genomic regions corresponding to the 51 selected differentially expressed or literature derived proteins (Table S1) were surveyed for mutations.

Out of the 1,109,321 sequencing reads generated, 22.63% mapped uniquely to the human genome and overlapped with the 303,936 bases associated with the 581 target regions. Of the generated bases, 69.3 million mapped to the target, corresponding to an average 228 times sequence coverage of the target. >99.9% of the target was covered by at least one read. The distribution of reads between the different samples was good in general (Table 1) however multiplex identifier 3 (MID 3) proved to have been very inefficiently amplified in the emPCR, as verified later by qPCR of the MIDs [23], and the sample labeled with MID 9 generated very low concentrations at the library preparation, which resulted in a lower molar amount of DNA from the MID 9 tagged sample in the pool.

**Table 1:** Distribution of mapped reads, bases, coverage and variants per sample.

Sample	Mapped reads	Mapped bases	Ave coverage	SNVs	Coding SNVs
MID1	35243	9.9mil	33	247	66
MID2	30264	8.6mil	28	215	50
MID3	2739	0.8mil	3	27	8
MID4	22952	6.4mil	21	217	55
MID5	30869	8.5mil	28	230	46
MID6	18608	5.2mil	17	221	63
MID7	20239	5.6mil	18	185	48
MID8	38539	10.7mil	35	252	53
MID9	7815	2.2mil	7	129	34
MID10	40046	11.4mil	38	259	65
Total	247314	69.3mil	228	1982	488

Distribution of mapped reads, bases, coverage and single nucleotide variants (SNVs) across the ten multiplex enriched breast cancer tumor samples. An even distribution across all samples except those tagged with multiplex identifier tag 3 and 9. Poor amplification characteristics of MID 3 was later verified by qPCR [21] to be the reason for poor representation of the corresponding sample among the sequence reads. The sample tagged with MID 9 was added in a lower molar amount upon pooling due to a low DNA concentration after library preparation.

In total 1,982 single nucleotide variations (SNVs) at 579 unique positions in the target regions were found in the tumor samples when compared to the human reference sequence (hg19). A higher rate of variation, per base, was seen in 5′UTR, 3′UTR, promotor and intron sequence of the target (0.07%), than in protein coding exon sequence (0.05%). Of the 149 unique positions with SNVs in protein coding sequence (488 SNVs total), 66 were subjected to an amino acid change. 15 of these non-synonymous alterations had not previously been reported in dbSNP build 130. All novel SNVs were detected in frequencies above 20% of the reads. Six of these non-previously reported variations were encountered in more than one tumor sample, and were confined to three genes, one in SATB1, four in MUC5AC and one in DDX26B (Table 2).

**Table 2:** Encountered single nucleotide variants (SNVs) overview.

Total unique positions with single nucleotide variants (SNVs)	579
In target genes	467
In exons (incl UTR)	266
In introns	201
In coding sequence (CDS)	149
In non-coding sequence	430
Non-synonymous in CDS	66
Novel non-synonymous not in dbSNP	15
Verified novel non-synonymous not in dbSNP	9
Novel non-synonymous present in more than one patient	6
**Verified novel non-synonymous present in more than one patient**	**2**
**Genes with verified novel non-synonymous present in more than one patient**	**SATB1(1), DDX26B(1)**

An overview of the single nucleotide variations encountered across the samples. 149 unique positions with single nucleotide variations (SNVs) were found in protein coding sequence, 15 of which gave rise to a different amino acid and were previously unreported in dbSNP (version 130). In total six of these turned out to be false positives when verified by bidirectional Sanger sequencing. Two of the remaining mutations were present in more than one individual and were located in the SATB1 and DDXB26 genes.

Confirmatory Sanger sequencing was carried out for all 15 novel SNVs in the tumor samples and normal reference tissue. This resulted in confirmation of nine of the 15 SNVs as heterozygous variations present in both tumor and normal tissue. The six remaining variations turned out to be false positives.

## Discussion

The percentage of reads mapping to the targeted regions for the used enrichment platform has previously been reported around 60–80% [19]. There are several possible reasons for the modest fraction of sequencing reads that mapped to the targeted regions in the present study. Variation in enrichment success can always be expected and can be coupled to the array-design, the sequence in the targeted regions, and the number and spread of the target regions across the genome. From these three aspects our approach was as difficult as possible. The design algorithm was the first version provided by the manufacturer, the locations of the selected target regions could be denoted as close to random and the target region length as relatively short (average length 521 bp). It is also feasible to believe that the multiplexing of samples on the capture array may have influenced the result with more non-targeted regions being able to remain close to the array surface through binding to the handles of real target fragments hybridized to the actual array.

To determine the usability of the results generated through the translational selection, multiplexed enrichment and sequencing methods, a high level of correlation to dbSNP for the non-synonymous SNVs should give a high validity to the method employed to find the variations. Additionally several of the non-synonymous SNVs (66 in total; Table 2) were present in genes with a previously established connection to breast cancer development through inherited genetic variations such as BRCA1 (seven SNVs) and BRCA2 (two SNVs) [24]. This together with high bidirectional sequence coverage should make the nine verified non-synonymous variations, present in normal and cancerous tissue in their respective individuals and not previously present in dbSNP (version 130), interesting for further analysis.

On the other hand, of the 15 non-synonymous mutations that were encountered in the samples, six turned out to be false positives when verified by bidirectional Sanger sequencing (Table S2). This raises increased concerns relating to the generation of systematic errors by present massive sequencing platforms. Further, comparison of these six seemingly novel non-synonymous mutations to the latest version of dbSNP (version 131) marks two of them as previously reported. Given previous false positive results already raising concerns to the quality of the content of dbSNP [25], this provides even further reason to exercise care when using the current versions of variant databases.

Two of the remaining previously unreported non-synonymous SNVs were found in the genomes of more than one individual. Each was verified as a heterozygous SNV present in both normal and cancerous tissue. Their presence in SATB1, previously linked to breast cancer promotion [26], and DDX26B, to our knowledge previously unreferenced in relation to breast cancer, provides further support for continued examination of the role of the genes in conjunction to breast cancer.

Lastly, the higher frequency of variations seen in non-coding sequence as compared to coding sequence has previously been reported in healthy [18] and cancerous [17] tissue, strengthening the scientific grounds for the logical reasoning that non-protein-coding regions of the genome are less subjected to evolutionary constraints.

In summary, the employment of a translational database selection strategy in combination with multiplexed enrichment by sequence capture provides a tool for careful biomarker discovery. Given the abundance of false positives generated by massive sequencing approaches, employing a rational selection strategy prior to sequencing can provide an efficient means to limit the number of variants at the end of the pipeline, enabling a complete variant verification process and a more reliable final list of biomarker candidates.

## Supporting Information

Table S1 **Selected genes.** 41 proteins and their corresponding genes were selected through the HPA database and 10 more proteins and their corresponding genes known to be associated with cancer from literature were added to the list.
(DOC)

Table S2 **Novel non-synonymous SNVs.** Among the novel non-synonymous SNVs encountered 40% turned out to be false positives. Two of the remaining SNVs were present in more than one individual.
(DOC)
