# Supplementary material for: Translational Database Selection and Multiplexed Sequence Capture for Up Front Filtering of Reliable Breast Cancer Biomarker Candidates
Source: PLoS One. 2011 Jun 15;6(6):e20794. doi: 10.1371/journal.pone.0020794 (PMC3115972; doi:10.1371/journal.pone.0020794)
Supplement: Table S1 — Selected genes. 41 proteins and their corresponding genes were selected through the HPA database and 10 more proteins and their corresponding genes known to be associated with cancer from literature were added to the list. (DOC) [file pone.0020794.s001.doc]

**Table S1. Selected genes**

| Database selected genes | | |
| --- | --- | --- |
| *Gene name* | *Ensembl ID* | *Description* |
| ACSS2 | ENSG00000131069 | Acetyl-coenzyme A synthesase, cytoplasmic. |
| ACTG1 | ENSG00000184009 | Actin, cytoplasmic 2 (Gamam Actin). |
| AUTS2 | ENSG00000158321 | Autism susceptibility gene 2 protein |
| BTN3A1 | ENSG00000026950 | Butyrophilin subfamly 3 member A1 precursor (CD277 antigen) |
| C1orf76 | ENSG00000143340 | UPF0417 protein C1orf76 |
| C9orf46 | ENSG00000107020 | Uncharacterized protein |
| C21orf104 | ENSG00000183412 | Uncharacterised protein. (fragment). |
| CDKN2A | ENSG00000147889 | Cyclin-dependent kinase inhibitor 2A, isoform 4 |
| CCR7 | ENSG00000126353 | C-C chemokine receptor type 7 precursor. |
| CD44 | ENSG00000026508 | CD44 antigen precursor. |
| CDH11 | ENSG00000140937 | Cadherin-11 precursor. |
| CHEK2 | ENSG00000183765 | Serine/Theronine-protein kinase Chk2 |
| CLPP | ENSG00000125656 | Putative ATP-dependent Clp protease proteolytic subunit |
| CST3 | ENSG00000101439 | Cystatin-C precursor (Cystatin-3) |
| DDX26B | ENSG00000165359 | Protein DDX26B |
| EPHB3 | ENSG00000182580 | Ephrin type-B receptor 3 precursor |
| FADD | ENSG00000168040 | Protein FADD (FAS-associated death domain protein) |
| GALNT6 | ENSG00000139629 | Polypeptide N-acetylgalactosaminyltransferase 6 |
| GLUL | ENSG00000135821 | Glutamine synthetase |
| GM2A | ENSG00000196743 | Ganglioside GM2 activator precursor |
| JUB | ENSG00000129474 | Ajuba isoform 1 |
| KPNA2 | ENSG00000182481 | Importin subunit alpha-2 |
| KIT | ENSG00000157404 | Mast/stem cell growth factor receptor precursor. |
| LGALS1 | ENSG00000100097 | Galectin-1 (Lectin galactoside-binding soluble 1) |
| LSR | ENSG00000105699 | Lipolysis-stimulated lipoprotein receptor. |
| MDK | ENSG00000110492 | MDK Midkine precursor (MK) |
| MPO | ENSG00000005381 | Myeloperoxidase precursor |
| MUC5B | ENSG00000117983 | Mucin-5B precursor |
| MYEOV | ENSG00000172927 | Myeloma overexpressed gene protein |
| MYOM2 | ENSG00000036448 | Myomesin-2 (M-protein) |
| NAGA | ENSG00000198951 | Alpha-N-acetylgalactosaminidase precursor |
| NAT1 | ENSG00000171428 | Arylamine N-acetyltransferase 1 |
| NPDC1 | ENSG00000107281 | Neural proliferation differentiation and control protein 1 precursor |
| PDPN | ENSG00000162493 | Podoplanin precursor. |
| PIP | ENSG00000159763 | Prolactin-inducible protein precursor. |
| RAB3D | ENSG00000105514 | Ras-related protein Rab-3D. |
| STK3 | ENSG00000104375 | Serine/threonine-protein kinase 3 |
| TFF1 | ENSG00000160182 | Trefoil factor 1 precursor (pS2 protein) |
| THAP7 | ENSG00000184436 | THAP domain-containing protein 7 |
| WNT9A | ENSG00000143816 | Protein Wnt-9a precursor (Wnt-14). |
| ZMYND11 | ENSG00000015171 | Zinc finger MYND domain-containing protein 11 |
| Literature selected genes | | |
| *Gene name* | *Ensembl ID* | *Description* |
| CRABP2 | ENSG00000143320 | Cellular retinoic acid-binding protein 2 |
| CCND1 | ENSG00000110092 | G1/S-specific cyclin-D1 (PRAD1 oncogene) (BCL-1 oncogene) |
| SATB1 | ENSG00000182568 | DNA-binding protein SATB1 |
| SATB2 | ENSG00000119042 | DNA-binding protein SATB2 |
| BRCA1 | ENSG00000012048 | Breast cancer type 1 susceptibility protein |
| BRCA2 | ENSG00000139618 | Breast cancer type 2 susceptibility protein |
| P65/RELA | ENSG00000173039 | Transcription factor p65 (Nuclear factor NF-kappa-B p65 subunit) |
| PTEN | ENSG00000171862 | Phosphatidylinositol-3,4,5-trisphosphate 3-phosphatase |
| STK11 | ENSG00000118046 | Serine/threonine-protein kinase 11 |
| P53 | ENSG00000141510 | Cellular tumor antigen p53 (Tumor suppressor p53) |
