# Supplementary material for: Translational Database Selection and Multiplexed Sequence Capture for Up Front Filtering of Reliable Breast Cancer Biomarker Candidates
Source: PLoS One. 2011 Jun 15;6(6):e20794. doi: 10.1371/journal.pone.0020794 (PMC3115972; doi:10.1371/journal.pone.0020794)
Supplement: Table S2 — Novel non-synonymous SNVs. Among the novel non-synonymous SNVs encountered 40% turned out to be false positives. Two of the remaining SNVs were present in more than one individual. (DOC) [file pone.0020794.s002.doc]

**Table S2. Novel non-synonymous SNVs**

| Verified | Gene | SNV position (hg19) | Present in individual |
| --- | --- | --- | --- |
| Positive | CDH11 | chr16:65005933 | 8 |
| Positive | CDH11 | chr16:65016164 | 8 |
| Positive | DDX26B | chrX:134713928 | 2, 8 |
| False positive | EPHB3 | chr3:184294914 | 1 |
| False positive | KIT | chr4:55602733 | 1 |
| Positive | MPO | chr17:56355393 | 4 |
| False positive | MUC5AC | chr11:1253975 | 4, 5, 6, 7, 8, 10 |
| False positive | MUC5AC | chr11:1253979 | 4, 5, 6, 7, 8, 10 |
| Positive | MUC5AC | chr11:1254317 | 2 |
| False positive | MUC5AC | chr11:1258196 | 1, 2, 5, 6, 7, 8, 10 |
| False positive | MUC5AC | chr11:1258239 | 1, 5, 6, 7, 8, 10 |
| Positive | MYEOV | chr11:69063475 | 5 |
| Positive | MYOM2 | chr8:2054076 | 10 |
| Positive | PIP | chr7:142836645 | 4 |
| Positive | SATB1 | chr3:18392818 | 2, 10 |
